# Insanity in the Lower Animals

**Published:** 1882-10

**Authors:** 


### EDITORIAL DEPARTMENT.

### INSANITY IN THE LOWER ANIMALS.

THE late Dr. Lindsay, author of "Mind in the Lower Animals," was a very enthusiastic expounder of the doctrine that all animals had some mind, including reason and consciousness, and that not a few were quite the superiors of men. In one of his volumes he calls attention to the subject of mental diseases in lower animals, and thinks that veterinarians could not be better employed than in studying this hitherto neglected department of pathology. Animal insanity is much more frequent than is supposed. It will explain many things in animal behavior often attributed to stupidity or viciousness.

The forms of the disease which are to be looked for are:

1. Idiocy and imbecility, which are simply different degrees of a congenital weak-mindedness.

2. Dementia, a loss of mind in the previously intelligent.

3. Mania, which is a term for madness in general. It may be (*a*) acute, or raving mania; (*b*) chronic; (*c*) it may be of a depressed form, when it is called *melancholia*.

4. Monomania of various forms may exist. Thus the animal may have a mania of suspicion, or for biting, or suicide, or a sexual mania, erotomania. The disease may chiefly affect the moral nature, and the animal become violently vicious.

1. Regarding idiocy, it is described by Pierquin. Darwin refers to imbecility in a dog, and describes its senseless habit of rotation or gyration. This is a common phenomenon of insanity in that animal. It is important to recognize imbecility if it exists, since much labor in attempts to train and educate animals may thus be spared.

2. Dementia is brought on in the horse, mule, cat, dog, etc., by blows on the head, passion, fright, meningitis and encephalitis. Pierquin describes a lively and intelligent parrot demented by the fright of a naval battle. Dr. Major asserts that the pathological changes (atrophy and degeneration) in dementia in the dog resemble those found in man.

3. Acute mania is often recognized. It should not be confounded with rabies, of which mania is only one symptom, and that not uniformly present.

Acute mania is generally ascribed by veterinary authors to acute inflammation of the brain or membranes, but this view is certainly not always correct.

It cannot always be easy to distinguish acute mania in animals from exaggerated passion.

(*a*) In chronic mania the mind, though disordered, has only occasional periods of excitement. The rogue elephants of India are assigned to this category. Baker describes a case occurring in a bull hippopotamus. The occurrence of chronic mania in the domestic animals is not rare, but as they are generally soon killed, little opportunity for studying or recording their symptoms is given.

(*b*) Melancholia is a form of disease to which some animals are predisposed. Such are the loris and certain monkeys. Homesickness is sometimes a cause of melancholia in such animals as the cat and dog.

(*c*) The various forms of monomania such as erotomania, kleptomania, dipsomania (e. g., Jumbo?), moral insanity, are discussed by Dr. Lindsay, who affirms that their distinguishing characters are easily made out.

